# Visualization of anisotropic-isotropic phase transformation dynamics in battery electrode particles

**DOI:** 10.1038/ncomms12372

**Published:** 2016-08-12

**Authors:** Jiajun Wang, Yu-chen Karen Chen-Wiegart, Christopher Eng, Qun Shen, Jun Wang

**Affiliations:** 1 Photon Science, National Synchrotron Light Source II, Brookhaven National Laboratory, Upton, New York 11973, USA

## Abstract

Anisotropy, or alternatively, isotropy of phase transformations extensively exist in a number of solid-state materials, with performance depending on the three-dimensional transformation features. Fundamental insights into internal chemical phase evolution allow manipulating materials with desired functionalities, and can be developed via real-time multi-dimensional imaging methods. Here, we report a five-dimensional imaging method to track phase transformation as a function of charging time in individual lithium iron phosphate battery cathode particles during delithiation. The electrochemically driven phase transformation is initially anisotropic with a preferred boundary migration direction, but becomes isotropic as delithiation proceeds further. We also observe the expected two-phase coexistence throughout the entire charging process. We expect this five-dimensional imaging method to be broadly applicable to problems in energy, materials, environmental and life sciences.

## Abstract (web summary)

Spatiotemporal phase evolution during transformations impacts materials performance in many systems. Here the authors use lithium iron phosphate particles inside a custom-designed battery to characterize in three dimensions the two-phase configuration at various states of charge via X-ray tomography.

## Graphical abstract

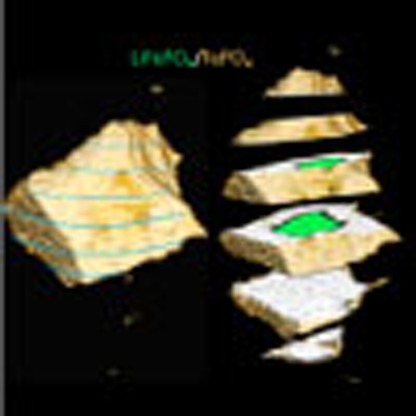

Direct observation of anisotropic (isotropic) phase transformation and mapping phase evolution is of importance in design and optimization of functional materials. X-ray tomography allows for characterization of the three-dimensional (3D) internal structure of large-volume structures^1^,^4^. This technique has long been used in life, medical and earth sciences to deliver 3D morphology information at micron-scale resolution^5^,^6^. In recent years, lens-based full-field transmission x-ray microscopy (TXM) with nanotomography capability has seen growing use in studying energy-storage materials^7^,^8^,^9^,^10^. Further progress in observing intermediates and chemical phases responsible for materials' performance requires quantitative measurement methods combining chemical sensitivity and high spatial resolution.

X-ray absorption near-edge structure (XANES) spectroscopy is sensitive to chemical and local electronic change of the probed element, and has been extensively exploited in characterizing fine structural change in a large variety of materials^11^,^12^. In combination with TXM, XANES enables mapping and tracking of chemical evolution under *in situ* conditions^13^,^14^,^15^,^16^,^17^. Nevertheless, the *in situ* XANES mapping approach has largely been restricted to two-dimensional (2D) observation as the obtained signal is usually spatially integrated along depth direction. For anisotropic phase transformations, quite common in technologically important materials, the method is limited in its ability to accurately capture phase evolution. Although tomographic scans at dual (below and above the adsorption edge of the studying element) and multiple energies have been achieved to identify the chemical element distribution^18^,^19^, it remains very challenging to carry out *in situ* studies on energy-storage materials, which requires accurately tracking chemical phase evolution in 3D with nanoscale resolution and correlating it to electrochemical performance. To pursue such studies using XANES requires reliable collection of multiple images over a 180°-rotation range at each energy point with sufficient energy resolution with the energy scanned across the absorption edge of the element of interest to produce a spectrum for each voxel of the sample inside a working electrochemical cell. Such an undertaking poses numerous technical and experimental difficulties.

Here, using full-field hard X-ray microscopy, we demonstrate an implementation of *in situ* XANES nanotomography able to build five-dimensional (5D) data sets tracking phase evolution in lithium iron phosphate particles in a working lithium-ion battery. Olivine lithium iron phosphate (LiFePO_4_) was selected as a model material because of its well-known two-phase process and representative behaviours for many energy materials^20^. Many-particle scale intercalation behaviour and atomic-scale phase transformation behaviours have been discussed in previous reports, but 3D features of single-particle phase evolution are yet to be fully established^21^,^22^. Figure 1 illustrates the basic principle of our approach, which is demonstrated using our full-field TXM with recently developed automated markerless tomography capability^23^. One specific feature of the setup is a built-in run-out correction system which enables automated tomography. First, this eliminates the need for a marker mounted on the sample or a special feature inside of the sample, enabling a wider range of samples to be studied and easier sample preparation. Second, manual alignment of hundreds of 2D projection images for 3D reconstruction becomes unnecessary, facilitating increased 3D spatial resolution through rapid collection of many projections and enabling time-resolved studies. Another important feature of the setup is that the image distance, the distance of the CCD detector to the zone plate lens, is automatically adjustable as a function of energy, which ensures that optimal resolution is preserved throughout energy scans. Together, these features make it practically feasible to combine XANES and nanotomography to study *in situ* phase transformations in 3D at nanoscale resolutions.

## Results

### Principle of XANES tomography

**Figure 1:**
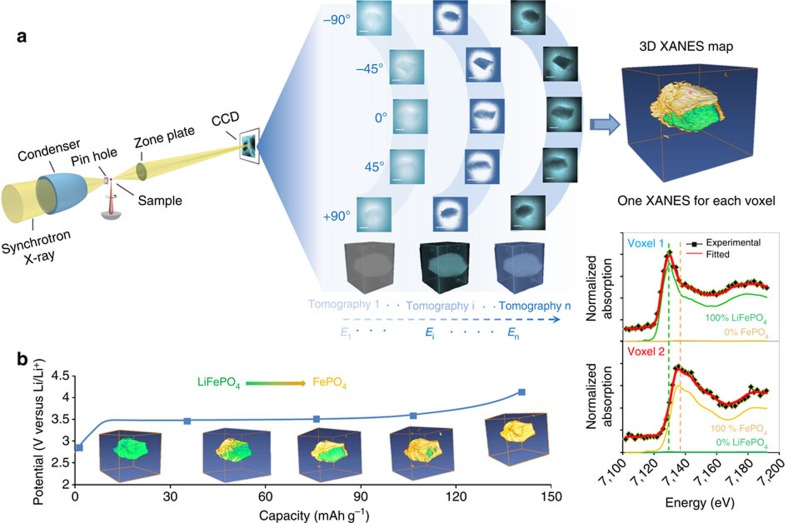
5D XANES tomography. (**a**) Schematic of experimental setup. A tomography data set spanning −90° to +90° is collected at each photon energy step (7,102 to 7,192 eV, 2 eV per step) across the near absorption K-edge of iron to produce chemical information for each voxel. By fitting the resulting spectra as a linear combination of spectra of end-phases, phase composition can be assigned to each voxel (shown in the right side of panel). (**b**) Chemical phase distribution as a function of capacity (or equivalently, time).

**Figure 2:**
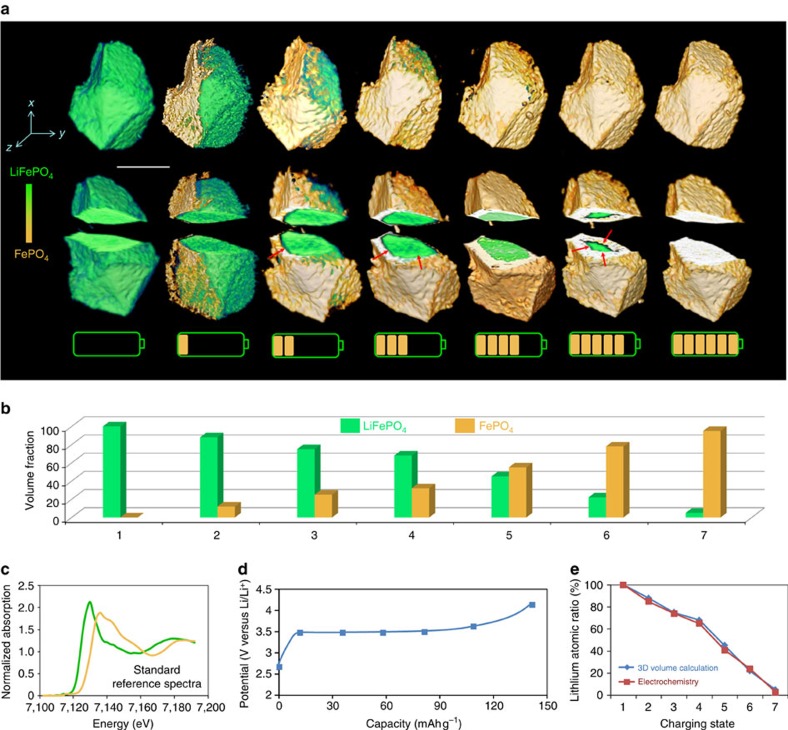
Chemical phase evolution. (**a**) Phase distribution as a function of charging time. The cut-away views reveal a change from anisotropic to isotropic phase boundary motion. (**b**) Phase volume fraction obtained from 3D quantitative analysis. (**c**) Standard XANES spectra for LiFePO_4_ and FePO_4_, showing clear energy shift of Fe K-edge. (**d**) The charging profile of LiFePO_4_ battery. XANES tomographic data sets were collected at the points indicated by the blue rectangles. (**e**) Agreement between lithium atomic ratio obtained from electrochemical measurement and from 3D volume analysis. Scale bar, 10 μm.

To acquire 3D XANES maps under *in situ* electrochemistry conditions, we designed a very compact half-cell fitting inside a quartz capillary, which enables us to record 2D projections over a complete angular range of 180° (see Supplementary Figs 1–2, Supplementary Movie 1) to ensure quality of 3D reconstructed structure. Figure 1 shows the basic principle of XANES tomography. At each charging stage (equivalently, point in time), a full series of 2D images were collected at each energy point to reconstruct the 3D structure, and a total of 46 energy points data sets were recorded across the near absorption K-edge of iron element (photon energy ranges from 7,102 to 7,192 eV with 2 eV per step). These 46 energy-dependent tomography data sets combined result in a 256 × 256 × 256 voxels 3D XANES data, where each voxel contains its own XANES spectrum. Each XANES spectrum of 256 × 256 × 256 voxels was then fitted as a linear combination of the bulk spectra of two standard olivine phosphate phases (LiFePO4 and FePO4) for all voxels, yielding a 3D map of the phase distribution over the sample volume (see Supplementary Figs 3–6). By repeating above procedure as charging proceeds, 3D chemical phase transformation evolution as a function of charging time can be rendered, as shown in Fig. 2.

### 5D chemical phase evolution

At the beginning of charging, phase boundary propagation is anisotropic with preferred paths, aligned imperfectly along a certain direction (Fig. 2a). The two-phase boundary moves primarily along the *y*-axis and then has an inclination toward *z*-axis, so a curved rather than flat phase interface is observed, as presented in cut-away view in Fig. 2a. To provide more detail on the anisotropic phase transformation, 3D morphology of the micron-sized particle at an earlier stage under charging is shown in Supplementary Fig. 7 and Supplementary Movies 2–5. The projected views and the corresponding 3D morphologies reveal a strong dependence on directions, which is associated with anisotropic lithium-ion diffusion. More details are clearly demonstrated in 3D morphologies shown in the two split-phase images in Supplementary Fig. 7.

With further charging, the particle becomes more delithiated and the phase boundary is seen to move along more directions and presents relatively isotropic features. This complex phase transformation behaviour is related to lithium-ion diffusion and misfit strain within solids^24^,^25^. In micron-sized particles, the ratio of surface energy to elastic energy is low, and misfit strain of the olivine phases can be relieved by the formation of cracks or structural dislocations^17^,^26^. Consequently, the weak driving force leads to phase boundaries propagating along preferential planes, producing the observed anisotropic behaviour at the beginning. As delithiation proceeds, the large lithium chemical potential gradient and growing internal strains may provide sufficient driving forces to move boundary propagation along multiple directions, producing the evolution towards isotropy as delithiation proceeds.

In addition to visualizing the evolution of phase distribution, our approach also allows quantitative determination, at particle scale, of phase composition by calculation of the 3D volume assigned to each phase. The determined volume fractions as a function of charging time are shown in the histograms in Fig. 2b, indicating a continuous phase transformation from LiFePO_4_ to FePO_4_ (the normalized Fe K-edge XANES spectra for standard LiFePO_4_ and FePO_4_ phases in Fig. 2c show a 2 eV energy shift, representing the classic two-phase transformation from Fe^2+^ to Fe^3+^), which is consistent with the state-of-charge obtained from the electrochemical charging protocol (Fig. 2d,e, Supplementary Fig. 8). The consistency between the 3D quantitative analysis and electrochemical performance measurement provides a validation of the *in situ* XANES mapping approach.

### Internal phase distribution in partially charged LiFePO_4_

**Figure 3:**
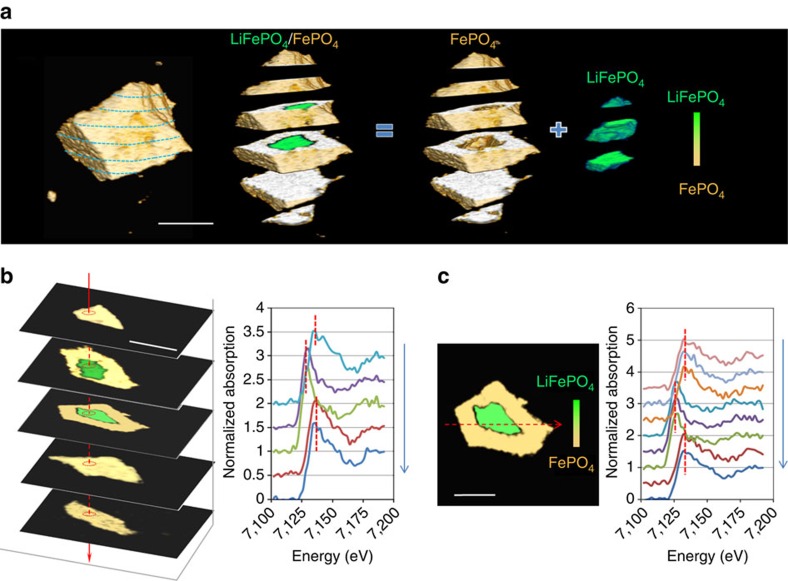
Sliced analysis of partially charged LiFePO_4_. (**a**) Sliced view of LiFePO_4_ at highly delithitated state. The sliced view reveals 3D internal core-shell phase distribution. (**b**) Cross-sectional slides and the corresponding XANES spectra along vertical axis. The spectra indicate the phase is either LiFePO_4_ or FePO_4_. (**c**) Line profile of XANES spectra along the horizontal axis at the selected cross-section slide. The spectra also indicated the chemical composition is either single LiFePO_4_ or FePO_4_ phase. Scale bar, 10 μm.

In addition to the anisotropic (and isotropic) phase transformation behaviours described, we observe two-phase coexistence within the LiFePO_4_ particle during the charging process. Particularly, when phase boundaries propagation changes from preferred directions (anisotropy) to multiple directions (isotropy) at highly delithiated state, a core-shell structure is clearly observed. A magnified cross-section view of LiFePO_4_ at a highly delithiated state is rendered in Fig. 3a, showing the core-shell internal structure with small amounts of the initial LiFePO_4_ phase (green) remaining inside. To quantitatively investigate the internal structure, a series of XANES spectra are extracted from the stack of slides vertically (Fig. 3b) and horizontally (Fig. 3c). Both stacks of spectra show a significant shift of about 2 eV (Fe K-edge) in energy, which is consistent with the standard spectra for LiFePO_4_ and FePO_4_, indicating that internal phase composition is either LiFePO_4_ or FePO_4_ with the absence of intermediate phases. Thus 3D XANES imaging of the internal structure provides further evidence that the classic two-phase coexistence for LiFePO_4_ persists at these reduced length scales.

## Discussion

**Figure 4:**
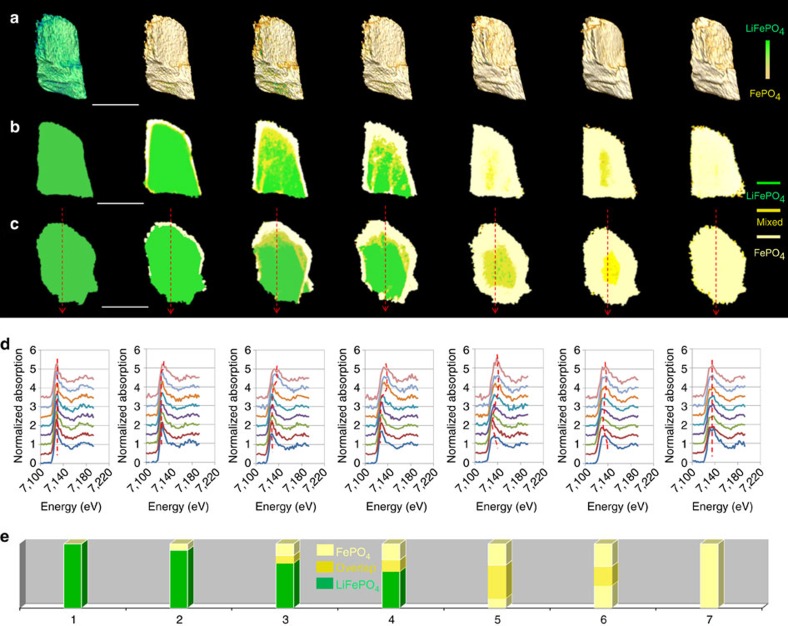
2D projections of full data sets. (**a**) 3D phase distribution evolution at a different visual angle from Fig. 2. From this view, it is difficult to make any conclusion about anisotropy/isotropy of phase transformation. (**b**,**c**) 2D projection XANES maps obtained from two different angles. These maps spatially compress chemical information, particularly for highly charged states where chemical phase identification becomes increasingly difficult. (**d**,**e**) 2D projection XANES and composition analysis obtained from the line profile in **c**. Some ‘intermediate' phases are detected, but are, in reality, artefacts resulting from the projected view. Scale bar, 10 μm.

The observed two-phase behaviour provides a concrete example for illustrating the limitations of 2D imaging approaches due to the unavoidable loss of chemical information along projection direction. Figure 4a shows the XANES tomography data for the same sample at a different view of angle, and as a comparison, the corresponding 2D projection maps at the two different orientations are shown in Fig. 4b,c. Large apparent deviation is observed between these 2D and 3D XANES maps. The 2D XANES maps in Fig. 4 appear to imply some ‘new'/‘intermediate' phases, especially for highly delithiated states (dark yellow colour in Fig. 4b,c and many consecutive irons K-edge energy shifts in Fig. 4d,e). A similar phenomenon was also observed in an *operando* 2D study (Supplementary Figs 9–12), which was performed on a similar sample in a conventional coin cell (the setup in Supplementary Fig. 9). Although ‘new'/‘intermediate' phases appear in these 2D XANES maps, it is hard to make any accurate chemical determination from the projected maps (Supplementary Figs 10–12). In reality, these ‘new' or ‘intermediate' phases are an artefact caused by the difficulty of uniquely interpreting complex 3D structures on the basis of projected images. As demonstrated here for the single-particle transformation in LiFePO_4,_ non-destructive XANES tomography avoids such difficulties, enabling more robust insight into internal and 3D evolution phase distribution during a chemical transformation.

Our work extends nanotomography to directly observe 3D phase transformation in solid-state materials as a function of both energy and time, revealing internal phase composition, evolving anisotropy of transformation and two-phase coexistence. Continuing advances in optics and growing availability of high-brightness synchrotron sources will allow this 5D chemical-imaging approach to capture faster processes accompanying electrochemical reactions and phase transformations. A result of particular interest is the correlation between 3D phase transformation and electrochemical capacity, showing correspondence between single-particle processes and performance which can be exploited in future efforts in materials development. Our methods for incorporating *in situ* electrochemical analysis can be potentially applied to other emerging imaging technologies such as lenseless coherent diffractive imaging to achieve higher spatial resolution, beyond X-ray optics resolution limits^26^,^27^,^28^,^29^.

## Methods

### Materials and electrode preparation

LiFePO_4_ powder, acetylene black and polyvinylidene fluoride (Sigma-Aldrich) with a weight ratio of 40:40:20 were mixed thoroughly in *N*-methyl-2-pyrrolidone solvent (Sigma-Aldrich), and the resultant slurry was pasted on commercial carbon paper (Toray Carbon Paper TGP-H-030). The electrode was dried under vacuum at 100 °C overnight and ready for use.

### Assembly of compact *in situ* electrochemical cells

The above LiFePO_4_ electrode was cut into a trapezoidal shape (∼40 μm short side, 800 μm long side and 1 cm height) under optical microscopy (LEICA DM4000M) with short edge to fit in the field of view (40 × 40 μm^2^) of the TXM. On the basis of the LiFePO_4_ loading of ∼0.6 mg cm^−2^, the trapezoid-shaped working electrode (∼0.045 cm^2^) contains ∼0.027 mg LiFePO_4_. Lithium foil and 1 M LiPF_6_ (dissolved in a solvent consisting of 50% ethylene carbonate and 50% dimethyl carbonate by volume) were used as the counter electrode and the electrolyte, respectively. The compact cell was fabricated in a quartz capillary (1 mm diameter) in an argon-filled glove box and sealed with epoxy. Two gold wires (0.5 mm diameter) connected the two electrodes to an external potentiostat to perform electrochemical measurement. As the LiFePO_4_ electrode was immersed completely in conventional electrolytes, this cell design is compatible with most conventional electrochemical measurements such as cyclic voltammetry and discharge/charge tests. The cells are stable enough to allow extended electrochemical cycling (over weeks) under x-ray characterization.

### *In situ* XANES tomographic data collection

The assembled cell was imaged using TXM at beamline X8C, National Synchrotron Light Source (Brookhaven National Laboratory, BNL). *In situ* electrochemical measurements were performed using a multichannel potentiostat (VMP3). Delithiation of LiFePO_4_ was galvanostatically carried out from an open-circuit potential to 4.2 V. Considering the large LiFePO_4_ particle size and low electronic/ionic conductivity, a low charge current corresponding to ∼0.01 C was used. XANES tomography data sets were collected at each stage of delithiation.

At each delithiation stage, energy scan ranges from 7,102 to 7,192 eV with 2 eV per step (46 energy steps in total). At each energy step, a tomography data set was collected using 361 projections over an angular range of 180° at a large field of view of 40 × 40 μm^2^ (binning 8 × 8 camera pixels into one output pixel). A total of 46 tomography data sets were collected at every 2 eV energy step across near absorption K-edge of iron, which enables the tracking chemical phases in each voxel of the sample throughout cycling.

Before reconstruction, raw data were corrected with a built-in run-out correction system which allows for automatic tomography alignment at beamline X8C.

### Tomographic XANES data analysis

Tomographic analysis of XANES data was carried out using customized software written in Matlab (MathWorks, R2011b) and developed in house at beamline X8C (NSLS, BNL). In all, 46 tomographic data sets were first reconstructed by standard filtered back-projection algorithm, yielding 46 reconstructed volumes for each time point. In combination with the corresponding tomographic slide at each energy step, a XANES data set was obtained for each voxel. A total of 256 × 256 × 256 XANES data sets were generated to map chemical information in 3D. On the basis of Beer's Law, the attenuation of x-rays by a given phase with attenuation coefficient, *μ* and thickness, *t* can be written as:


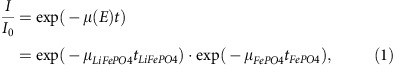


where, *I*_*0*_ is the incident x-ray intensity and *I* is the transmitted x-ray intensity. Notice that *μ* is a function of energy and can be attributed in our experiments to one of two phases: LiFePO_4_ and FePO_4_.

The scaled *−ln(I/I*_*0*_) at each of the 256 × 256 × 256 voxels was then fitted with the linear combination of two ‘pure-phase' *μ* values. The ratio of the weighting factor is analogous to the fraction of thicknesses of the phases, that is, to the volume ratio, as implied by,


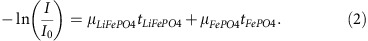


The fitting was carried out by minimizing the *R* value for each spectrum (for each voxel) with the definition,


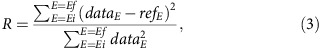


where, *E*_*i*_ is 7,102 eV, *E*_*f*_ is 7,192 eV, *data*_*E*_ is the *−ln(l/l*_*0*_) value for the given voxel at energy *E*, the ref_E_ is the reference *−ln(l/l*_*0*_) value, obtained from a linear combination of LiFePO_4_ and FePO_4_.

Standard reference XANES spectra were obtained measured using commercial materials, LiFePO_4_ (MTI Corp, USA) and FePO_4_ (Sigma, USA).

After the above linear combination fitting, 3D phase distribution (LiFePO_4_ and FePO_4_) at each delithiation step was retrieved. Following the same data analysis procedure, 5D visualizations (*x*, *y*, *z*, delithiation time and photon energy) of the phase transformation in LiFePO_4_ were rendered.

### *Operando* 2D TXM experiment

The *operando* 2D TXM experiment was performed on conventional 2,032 coin cells with Kapton windows^14^,^15^,^17^. Lithium metal was used as the counter electrode and 1 M LiPF_6_ in a mixture of ethylene carbonate/dimethyl carbonate (EC/DMC, 1:1 by volume) as the electrolyte. The above LiFePO_4_ electrode was used as the working electrode. To track phase transformation, charge–discharge characteristics at a potential range between open-circuit potential to 4.2 V (versus Li/Li^+^) was performed at room temperature. To study the chemical state evolution, a full XANES image series was collected at each charging stage during the delithiation process. Each XANES image series was measured by scanning Fe absorption K-edge from 7,092 to 7,192 eV, with 2 eV step size, and one TXM image at one energy step, which generated 1 k × 1 k XANES spectra with 2 × 2 binned pixels. Each image was collected with 10 s exposure time. Camera pixels (2 × 2) were binned into one output image pixel.

### Data availability

The data that support the findings of this study are available from the corresponding author on request.

## Additional information

**How to cite this article:** Wang, J. *et al.* Visualization of anisotropic-isotropic phase transformation dynamics in battery electrode particles. *Nat. Commun.* 7:12372 doi: 10.1038/ncomms12372 (2016).

## Supplementary Material

Supplementary Figures Supplementary Figures 1-12

Supplementary Movie 1 2D projections over 180 degree range showing irregular shape of LiFePO^4^ particle

Supplementary Movie 2 3D chemical phase distribution showing core-shell morphology in partially delithiated LiFePO^4^ particle

Supplementary Movie 3 Inner LiFePO^4^ core in partially delithiated LiFePO^4^ particle

Supplementary Movie 4 Outer shell of FePO^4^ in partially delithiated LiFePO^4^ particle

Supplementary Movie 5 Cut-away view of 3D chemical phase distribution in partially delithiated LiFePO^4^ particle
