# Supplementary material for: Visualization of anisotropic-isotropic phase transformation dynamics in battery electrode particles
Source: Nat Commun. 2016 Aug 12;7:12372. doi: 10.1038/ncomms12372 (PMC4990630; doi:10.1038/ncomms12372)
Supplement: Supplementary Figures — 1-12 [file ncomms12372-s1.pdf]

Supplementary Figures

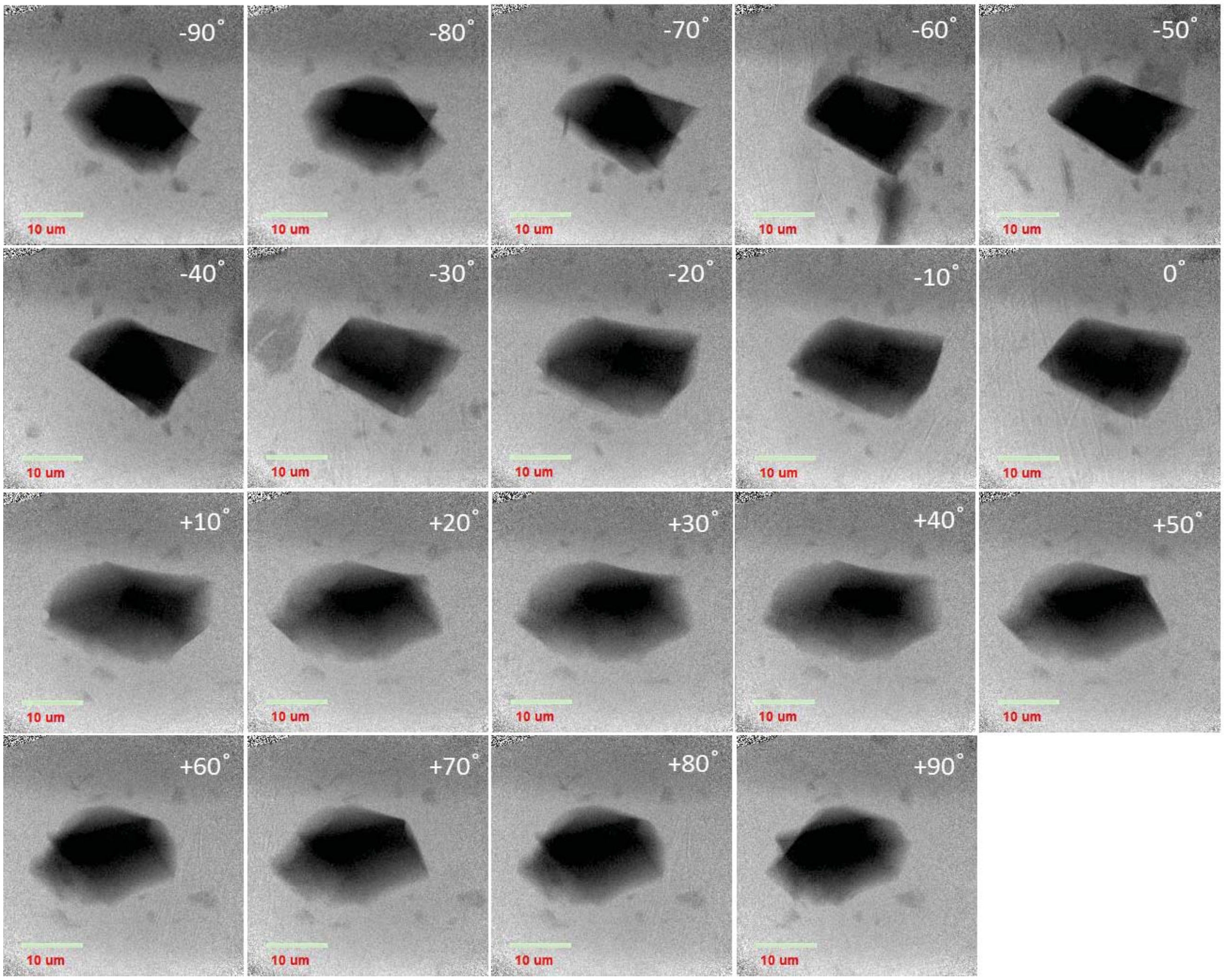

Figure S1. Selected 2D projections (-90˚ to +90 ˚) of the pristine sample at photon energy of 7192 eV (80 eV above K edge of iron). The particle shows different morphologies from different view of angles. It is hard to tell the true surface features in this particle without 3D tomography technique.

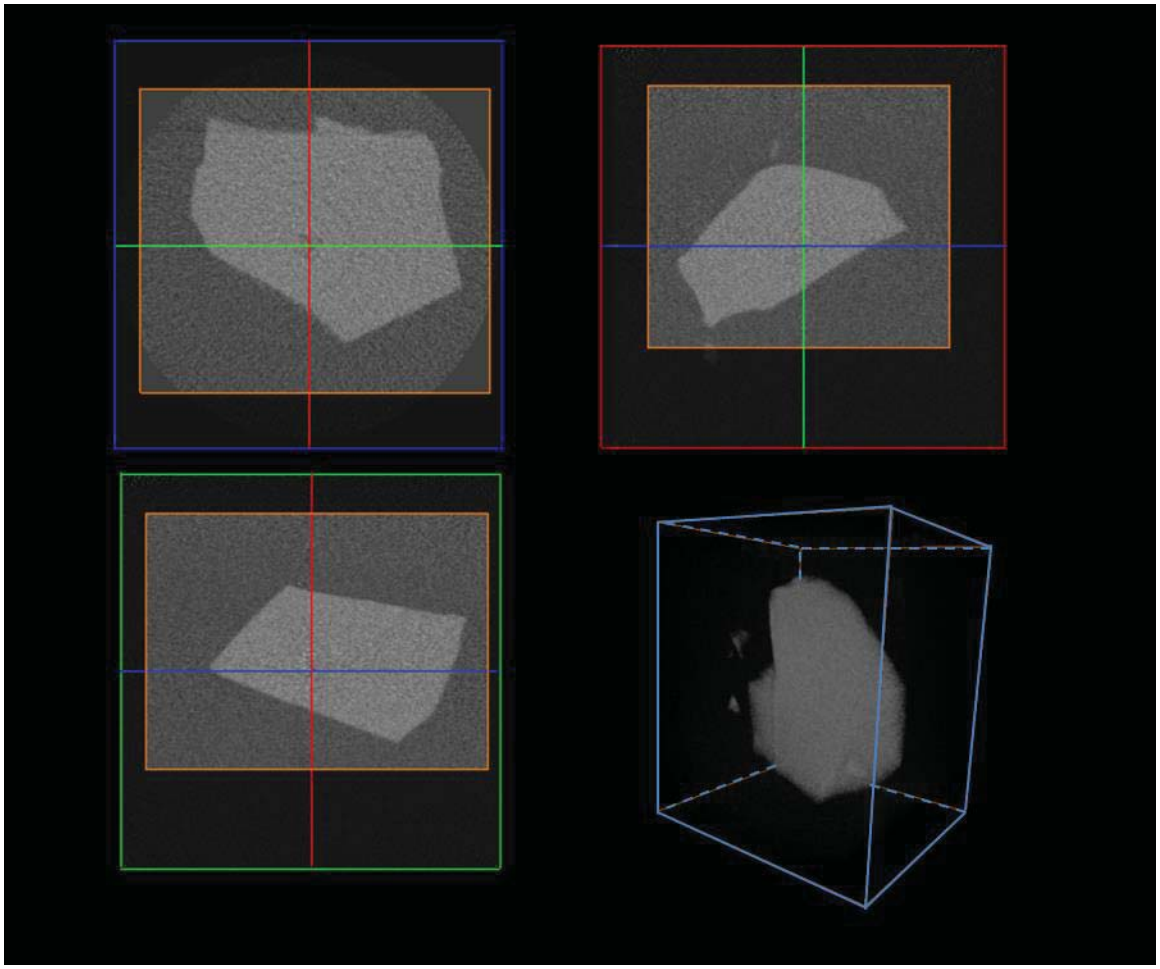

Figure S2. 3D reconstruction of $LiFePO_4$ particle at 7192 eV. These images reveal the irregular shapes of the particle. The cross-sectioned images show homogeneous attenuation contrast at this fixed energy (7192eV), indicating that chemical composition information cannot be analyzed at a fixed energy. It is therefore necessary to tune energy across the absorption edge of an element of interest.

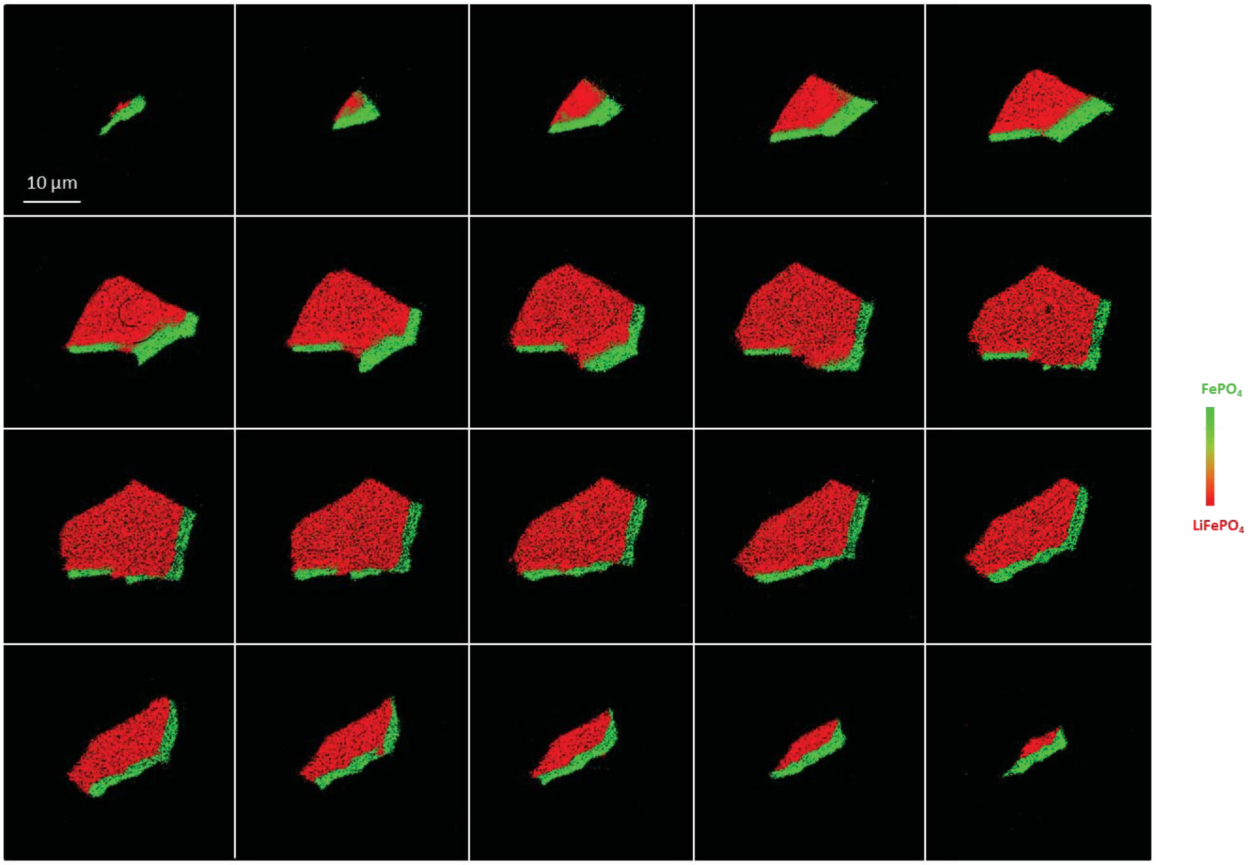

Figure S3. XANES maps at selected cross-sections of cathode particle at the initial charging stage.

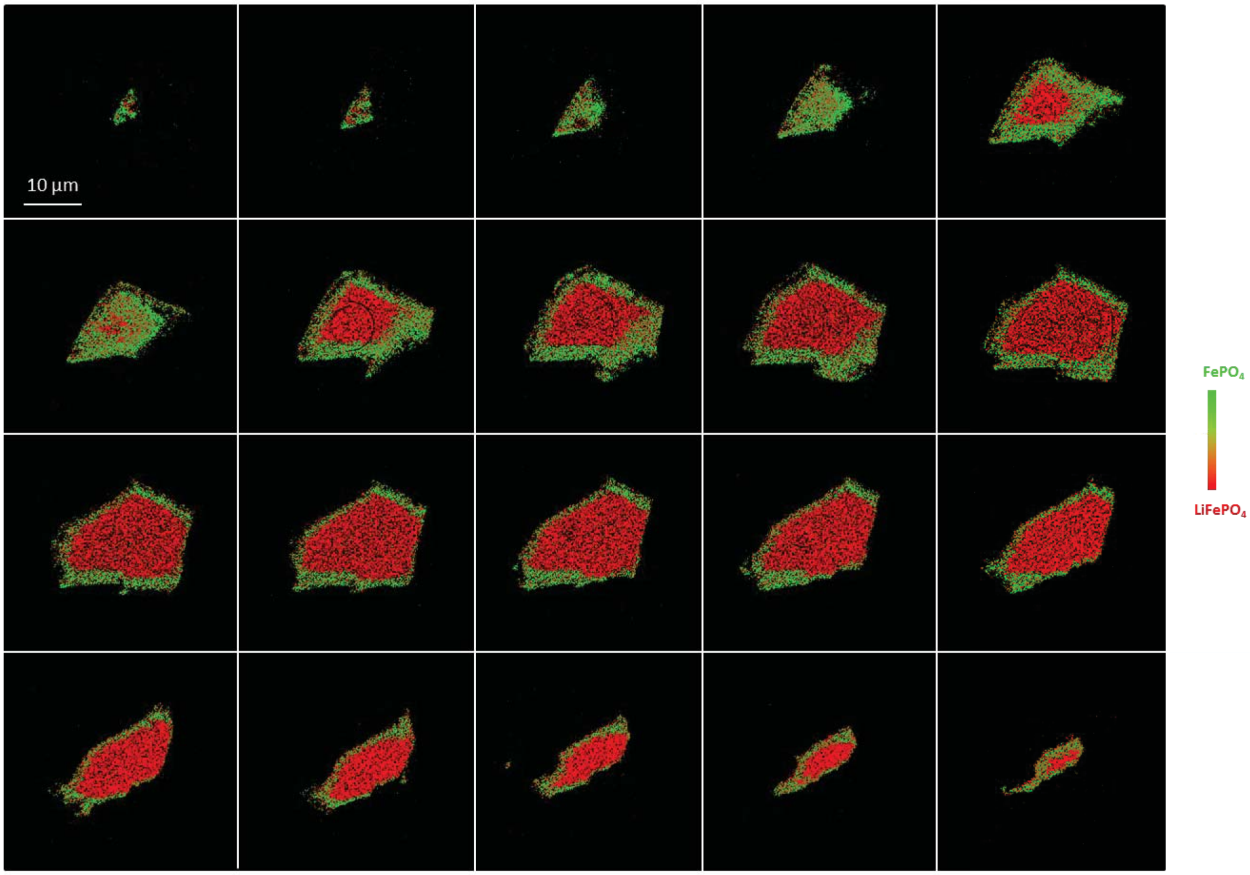

Figure S4. XANES maps at selected cross-sections of cathode particle at the partial charging stage.

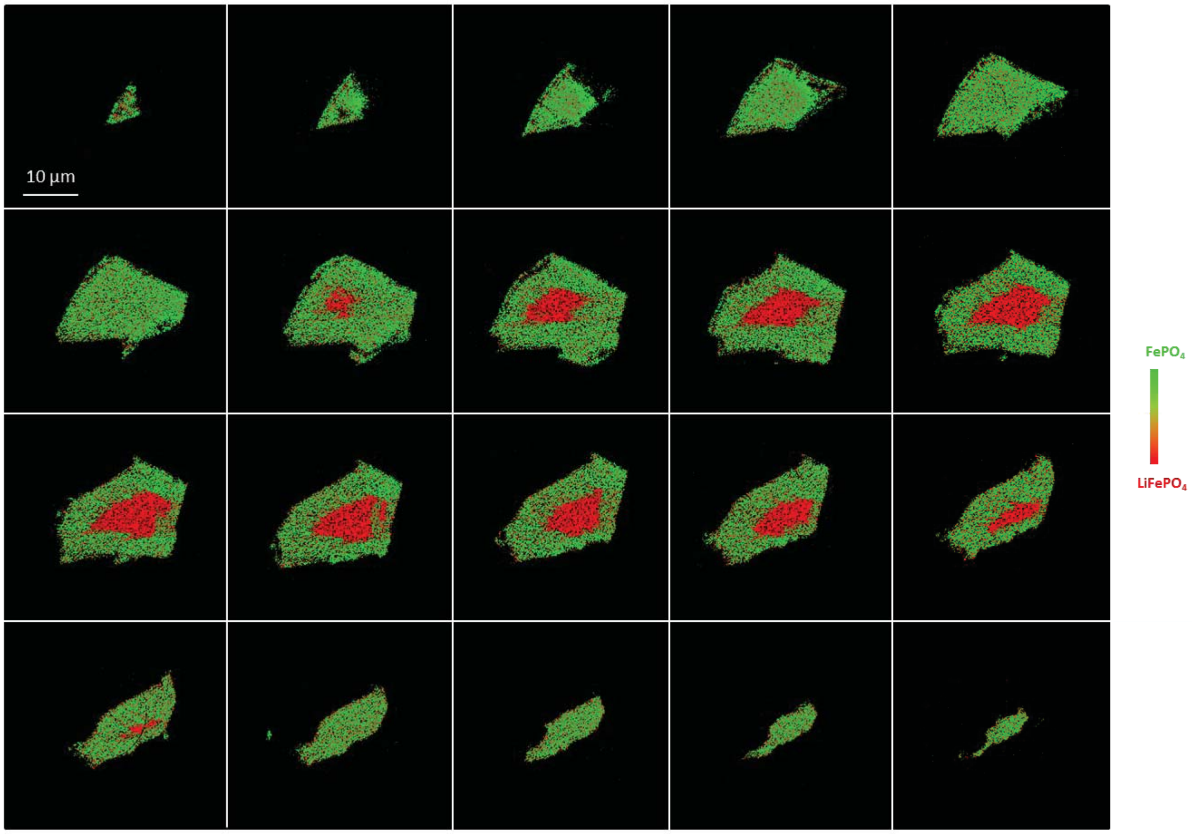

Figure S5. XANES maps at selected cross-sections of cathode particle at the middle charging stage.

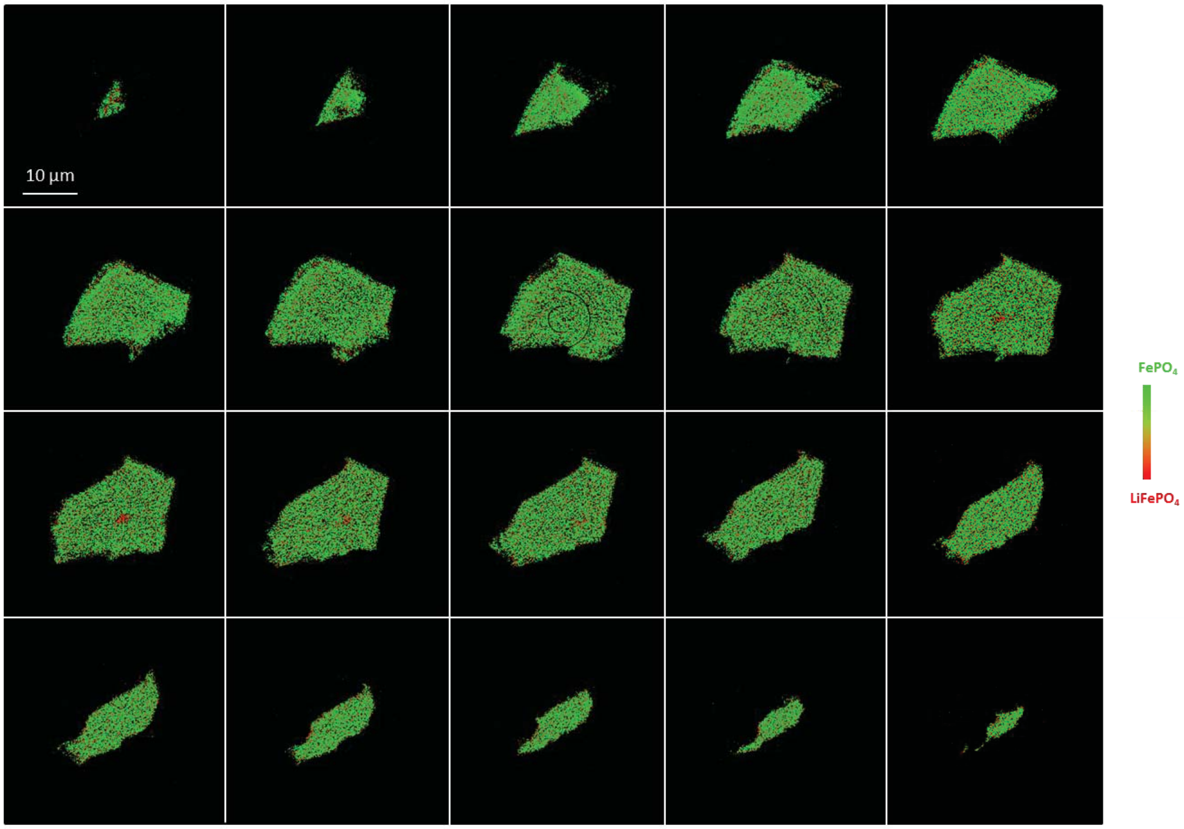

Figure S6. XANES maps at selected cross-sections of cathode particle at the end charging stage.

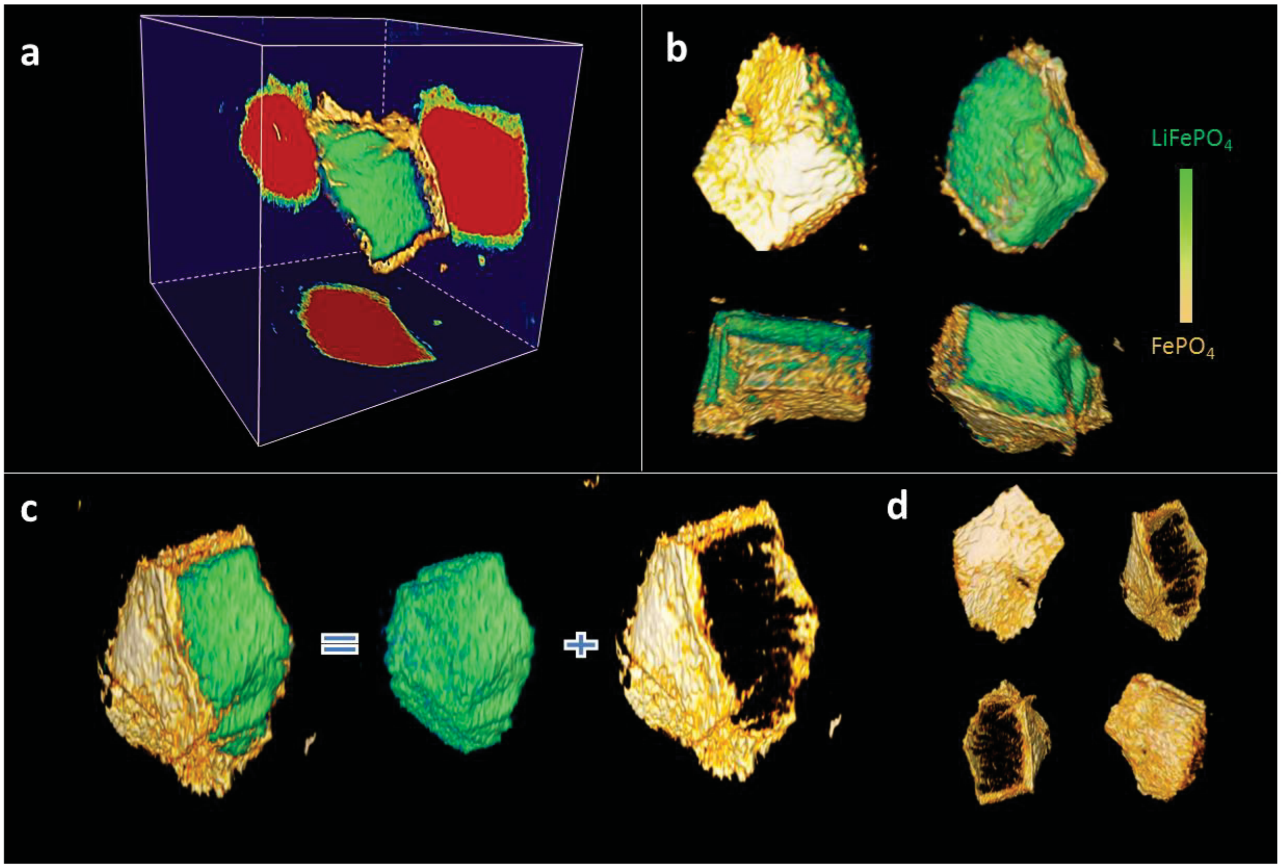

Figure S7. 3D tomography of partially delithiated sample. (a) the 3D overall morphology; (b) viewing along different orientations shows anisotropic phase transformation; (c) Split phases of $LiFePO_4$ (core, green) and $FePO_4$ (shell, golden); (d) viewing from different orientations for $FePO_4$ phase (shell, golden). These 3D phase distribution reveals that phase transformation in $LiFePO_4$ is anisotropic with preferred paths during the charging process.

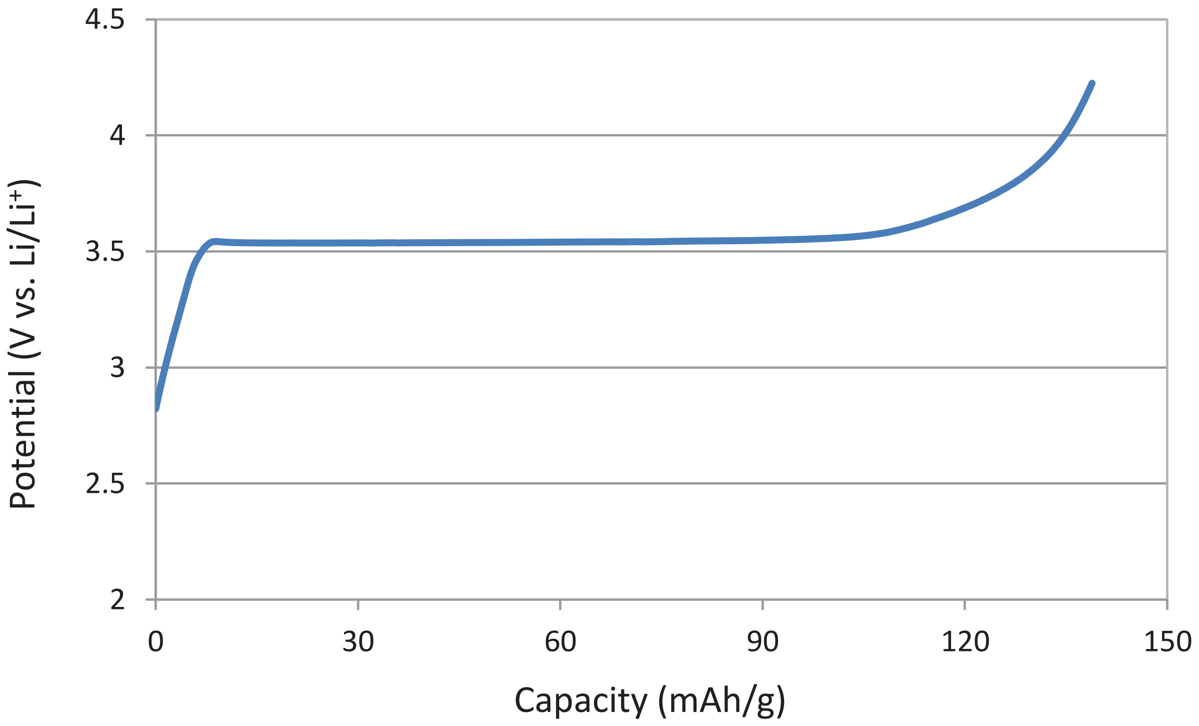

Figure S8. Charging profile for $LiFePO_4$ cathode. The flat charging voltage plateau at ~3.5V corresponds to a two-phase region. The obtained capacity (~140 mAh/g) is slightly lower than the theoretical value (~170 mA/g), which can be due to larger ion diffusion barrier in larger $LiFePO_4$ particle.

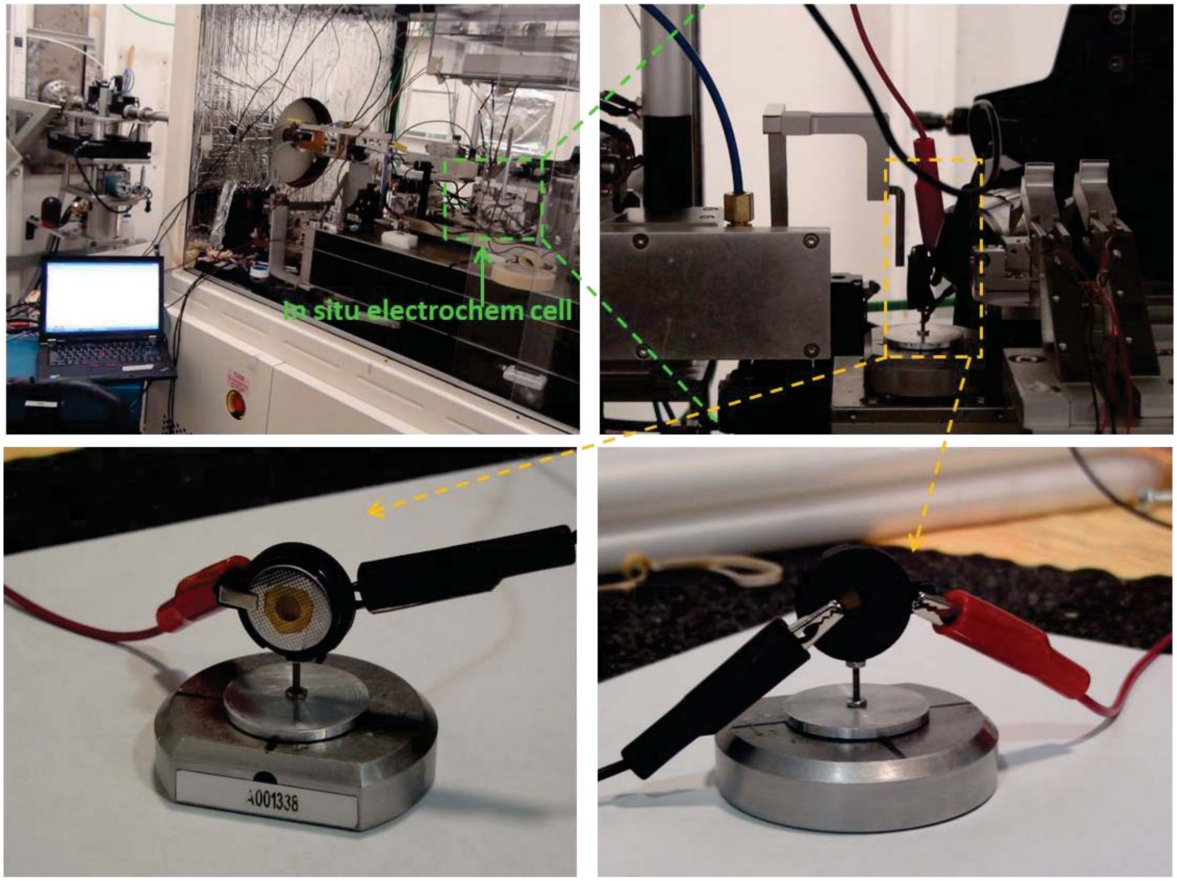

Figure S9. *In operando* 2D electrochemical cell setup. Conventional 2032 coin cells modified with two Kapton windows are used as an electrochemical cell for *in operando* 2D study. The two Kapton windows allow hard X-ray to go through the electrode. This conventional cell design is convenient to assemble in a glove box and can be applied in a variety of battery material study.

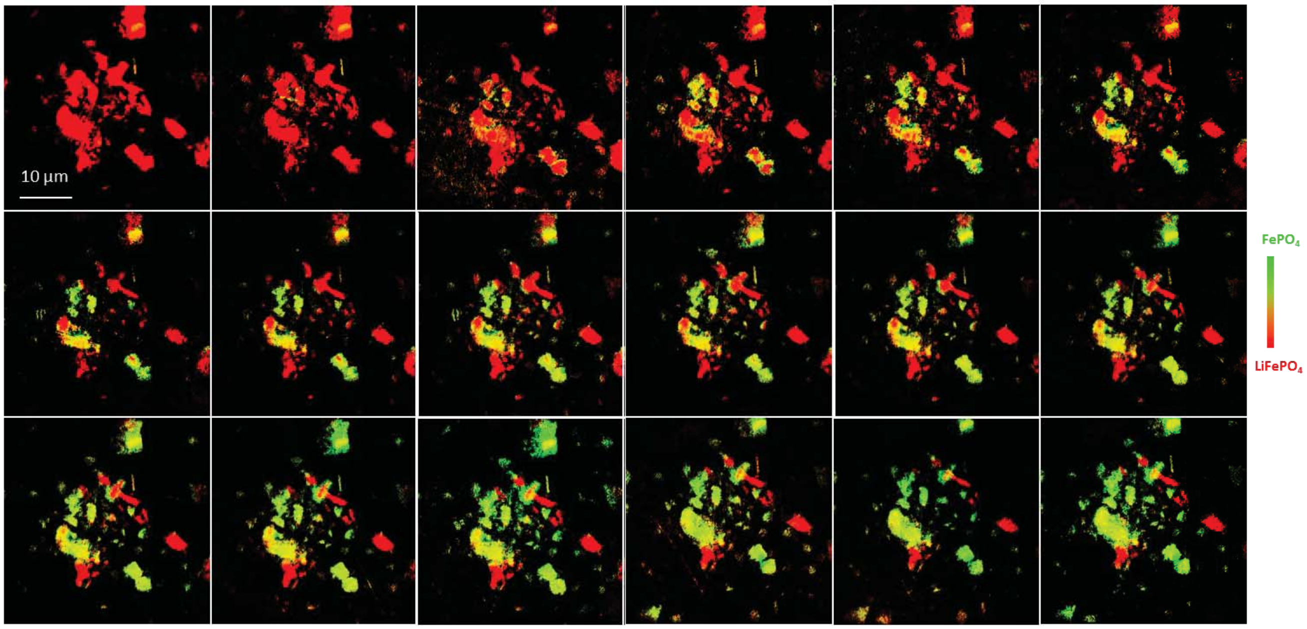

Figure S10. *In operando* 2D XANES imaging of $LiFePO_4$. The overwhelming majority of $LiFePO_4$ particles undergo phase transformation and many particles show mixed phase composition (yellow color) during charging (delithiation). Due to chemical information overlap at 2D projections, it is hard to accurately characterize the composition distribution from such images

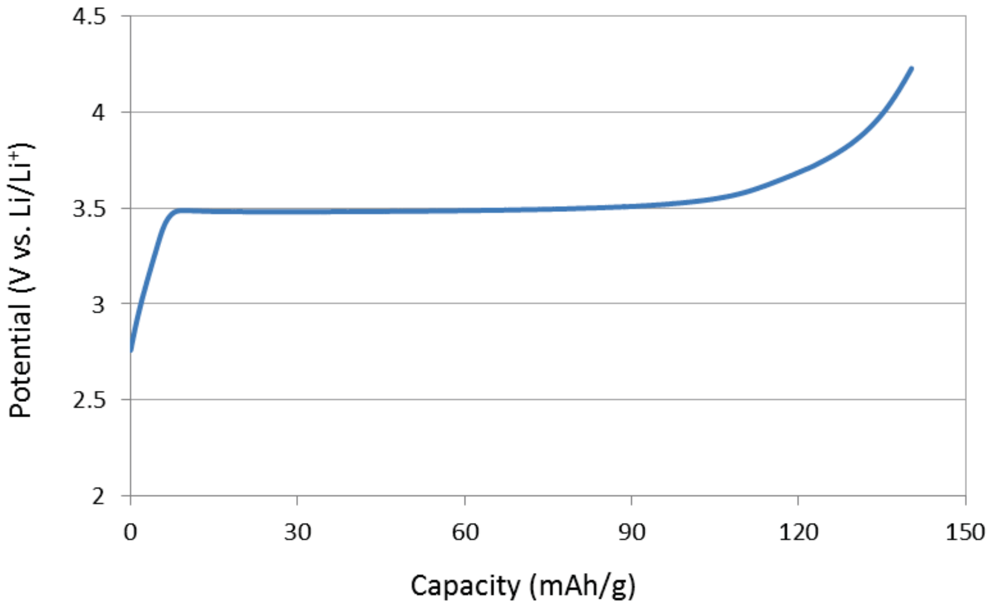

Figure S11. Charging profile for $LiFePO_4$ coin cell during *operando* 2D TXM experiment.

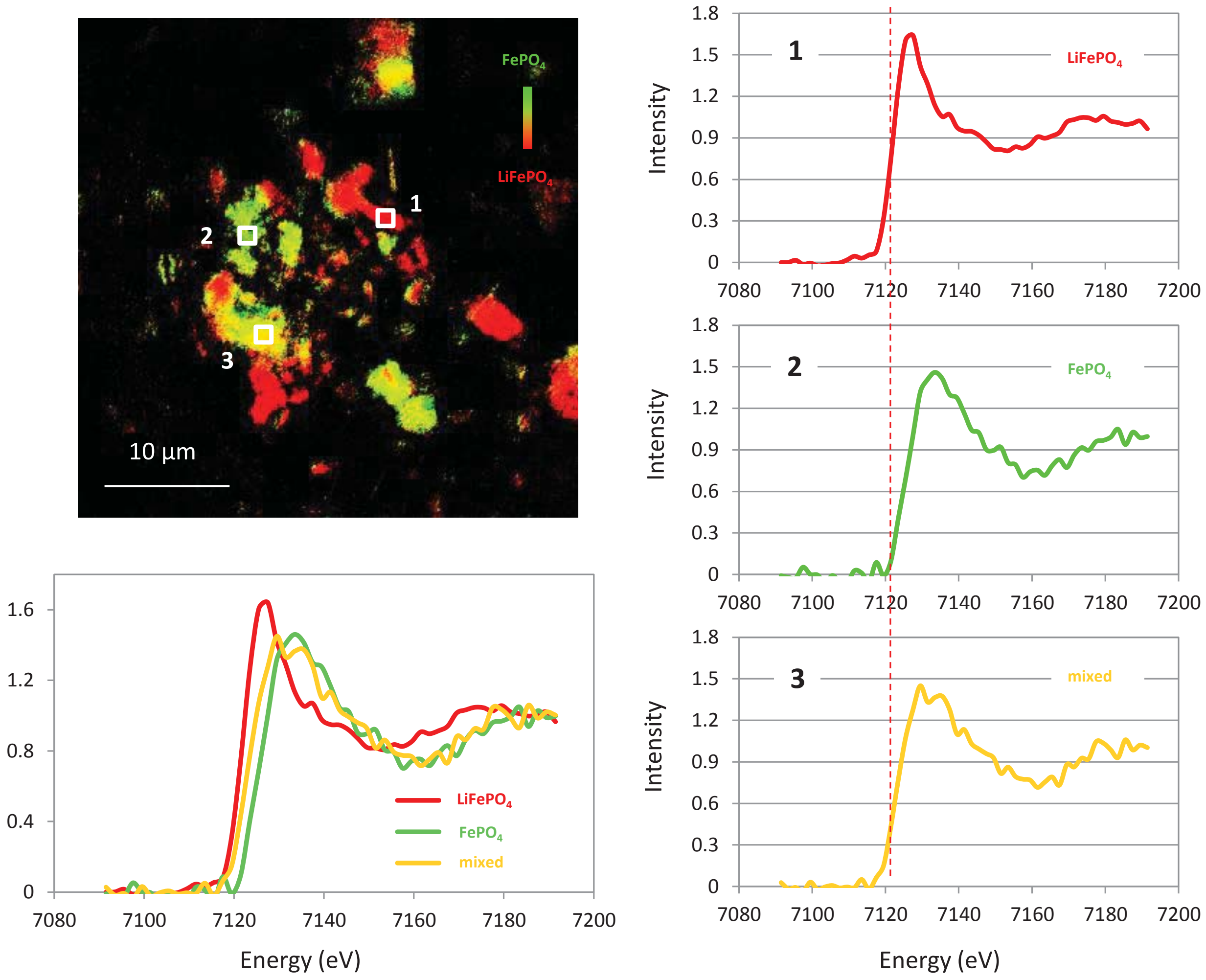

Figure S12. Phase composition analysis of partially delithiated $LiFePO_4$. A heterogonous mixture of phases is apparent in this 2D chemical map.
